# Effects of Yoga on Utero-Fetal-Placental Circulation in High-Risk Pregnancy: A Randomized Controlled Trial

**DOI:** 10.1155/2015/373041

**Published:** 2015-01-20

**Authors:** Abbas Rakhshani, Raghuram Nagarathna, Rita Mhaskar, Arun Mhaskar, Annamma Thomas, Sulochana Gunasheela

**Affiliations:** ^1^SVYASA University, 19 Eknath Bhavan, Gavipuram Circle, KG Nagar, Bangalore 560 019, India; ^2^St. John's Medical College and Hospital, Sarjapur Road, Bangalore 560 034, India; ^3^Gunasheela Surgical & Maternity Hospital, Building No. 1/2, Dewan Madhava Rao Road, Basavanagudi, Bangalore, Karnataka 560004, India

## Abstract

*Introduction*. Impaired placentation and inadequate trophoblast invasion have been associated with the etiology of many pregnancy complications and have been correlated with the first trimester uterine artery resistance. Previous studies have shown the benefits of yoga in improving pregnancy outcomes and those of yogic visualization in revitalizing the human tissues. *Methods*. 59 high-risk pregnant women were randomized into yoga (*n* = 27) and control (*n* = 32) groups. The yoga group received standard care plus yoga sessions (1 hour/day, 3 times/week), from 12th to 28th week of gestation. The control group received standard care plus conventional antenatal exercises (walking). Measurements were assessed at 12th, 20th, and 28th weeks of gestation. *Results*. RM-ANOVA showed significantly higher values in the yoga group (28th week) for biparietal diameter (*P* = 0.001), head circumference (*P* = 0.002), femur length (*P* = 0.005), and estimated fetal weight (*P* = 0.019). The resistance index in the right uterine artery (*P* = 0.01), umbilical artery (*P* = 0.011), and fetal middle cerebral artery (*P* = 0.048) showed significantly lower impedance in the yoga group. *Conclusion*. The results of this first randomized study of yoga in high-risk pregnancy suggest that guided yogic practices and visualization can improve the intrauterine fetal growth and the utero-fetal-placental circulation.

## 1. Introduction

Impaired placentation and fetoplacental hypoxia have been associated with the etiology of a number of pregnancy complications [1]. Proper placentation involves extensive vascular remodeling of the uteroplacental arteries, which play a major role in delivery of maternal blood to the intervillous space [2]. Failure of adequate trophoblast invasion to achieve this transformation of the spiral arteries has been associated with preeclampsia, preterm delivery, IUGR, and being small for gestational age [3, 4]. Conversely, it has been argued that improved uteroplacental and fetoplacental blood circulation could prevent these complications and also chronic diseases later in the life of the neonate [5]. The trophoblast invasion is completed by the 20th week of gestation [6]. It has been demonstrated that there is a close correlation between the first trimester uterine artery resistance and abnormal trophoblast invasion [6].

The word “yoga” is derived from the Sanskrit verb yuj, which means union. This refers to the union of the individual consciousness with that of the Universal Divine Consciousness that can be achieved by a wide variety of practices that range from certain postures (yoga asanas), breathing exercises (pranayama), hand gestures (mudras), cleansing exercises (kriyas), relaxation, and meditation techniques. The latter two include a wide range of practices, including visualization, guided imagery, and sound resonance practices. The rational for using these techniques requires a brief introduction on prana and its movements in the body.

The rationale for using techniques requires a brief introduction on prana and its movement in the body. According to the yogic sciences, beyond the physical body is the more subtle, pranic body, where the prana flows, and the mental body, where our thoughts are processed [7]. The frequency of our thoughts in the mental body influences the flow of prana in the pranic body, which in turn affects our health [7]. The idea of using visualization and guided imagery is to give order to our uncontrolled thoughts and in doing so regulate the flow of prana and improve the health of the physical organs. Consequently, it has been argued that visualization and guided imagery revitalize the tissues by activating the subtle energies (prana) within the body [8].

Being over 5000 years old, the science of yoga has been shown to impact a variety of physical and psychological health conditions, including anxiety, depression, metabolic syndrome, cancer, and cardiovascular, musculoskeletal, and pulmonary disorders [9, 10]. Additionally, yoga has been shown to improve the outcomes in low-risk [11] and high-risk pregnancies [12]. A study to investigate the effect of yoga in high-risk pregnancy was planned (funded by the Department of AYUSH, Ministry of Health and Family Welfare, Government of India) and the results showed significantly fewer pregnancy induced hypertension (PIH), preeclampsia, gestational diabetes (GDM), and intrauterine growth restriction (IUGR) cases in the yoga group (*P* = 0.018, 0.042, 0.049, and 0.05, resp.) and significantly fewer small-for-gestational-age (SGA) babies and newborns with low APGAR scores (*P* = 0.006) in the yoga group (*P* = 0.033) [12]. Ultrasound measurements of the fetal development and utero-feto-placental blood flow were also included in the same study. The present paper reports the effect of yoga on these parameters with the hypothesis that the benefits in high-risk pregnancy are due to improved placental blood flow after yoga. However, the sample sizes for the outcome paper [12] are not consistent with those of the present paper due to a slightly higher attrition rate in the Doppler data.

## 2. Methods

### 2.1. Sample-Size Calculations

Using the event ratios (0.185 in the experimental group and 0.506 in the control group) reported in a Japanese study, with *α* set at 0.05, probability of type I error at 0.01, powered at 0.8, a minimum sample size of 27 per group was obtained. As there were no published studies on yoga in high-risk pregnancies at the time of designing this study, we used the event ratios from the closest study by Kanako [13] on simple water exercises to prevent preeclampsia. We recruited a total of 93 subjects and the final analysis was made on 27 subjects in the yoga group and 32 in the control group.

### 2.2. Design and Settings

This was a randomized controlled prospective stratified single-blind trial. “Single-blind” refers to the fact that gynecologists, obstetricians, radiologists, and laboratory staff were blinded to the group selection. The trial was conducted at the Obstetric Unit of St. John's Medical College and Hospital (SJMCH) and Gunasheela Maternity Hospital (GMH) in Bengaluru, India.

### 2.3. Selection Criteria

*Inclusion Criteria*. Pregnant women within 12 weeks of gestation and with any of the following risk factors were considered qualified for the study: (1) history of poor obstetrical outcomes (pregnancy induced hypertension, preeclampsia, eclampsia, and intrauterine growth restriction); (2) twin pregnancies; (3) extremes of age: maternal age below 20 or above 35 years; (4) obesity: maternal body mass index of above 30; and/or (5) family history of poor obstetrical outcomes among blood relatives, that is, sister, mother, or grandmother. Groups were stratified at recruitment based on risk factors and the numbers were equal for each risk factor. However missing data during the study did not permit us to keep the groups matched for the analysis. Exclusion criteria: (1) Severe renal, hepatic, gallbladder, or heart disease; (2) structural abnormalities in the reproductive system; (3) hereditary anemia; (4) seizure disorders; (5) sexually transmitted diseases, or (6) any medical conditions that prevented the subject from safely and effectively practicing the interventions. While we did not exclude women with diabetes or essential hypertension, none of the participants enrolled in the study were ever diagnosed with these conditions prior to this pregnancy.

### 2.4. Recruitment and Randomization

Subjects within the 12th week of gestation were approached by a research staff at the reception of the Obstetrics Department of SJMCH or GMH and introduced to the project. Those who were interested were escorted by a staff to an annex room in the outpatient department itself, where the study was explained in detail, and then were screened using a written protocol. Qualified subjects were given the opportunity to sign the informed consent form in order to complete the recruitment and begin the randomization process. We used an online random number generator by GraphPad Software (www.graphpad.com/quickcalcs/randomize1.cfm, last accessed on June 16, 2013) to randomize a set of numbers into two groups. The selections (yoga or control) were then written on paper slips and placed in opaque envelopes, sealed, numbered, and kept in a locked cabinet. Recruited participants were assigned an ID and were permitted to pick one of the available envelopes to determine their group selection.

### 2.5. Ethical Clearance and Informed Consent

The Ethical Committee of SJMCH provided clearance for this study and approved its informed consent form before its commencement. All participants were required to sign this consent form in order to enroll in the study.

### 2.6. Interventions

The intervention set for each group was administered from the beginning of the 13th week to the end of the 28th week of gestation (a total of 28 sessions). The yoga group received standard care plus one-hour yoga session three times a week at the center and were instructed to practice the same routines at home. The control group received standard care plus walking for half an hour mornings and evenings (the routine antenatal exercise advised by the hospitals). The subjects in both groups were asked to keep a diary of their practices and daily physical activities, which was checked by the research staff during each of their visits to the antenatal department. The yoga classes were conducted by trained certified postgraduate yoga therapists, who used an instruction manual to conduct the classes at a reserved room within the premises of SJMCH/GMH. Standard care offered to both groups included the following: (1) pamphlets about diet and nutrition during pregnancy, (2) regular checkups by the obstetrician, and (3) biweekly follow-ups by the research staff. The purpose of these biweekly telephone follow-ups was to check if the subjects were adhering to their intervention practices and routine hospital check-ups.

The yoga intervention was selected very carefully from three categories: (1) yogic postures, (2) relaxation and breathing exercises, and (3) visualization with guided imagery. The yogic postures were chosen to reduce the physical side effects of pregnancy, such as edema, and strengthen the perineal muscles for delivery. The relaxation and breathing exercises were aimed at reducing the maternal stress. The visualization with guided imagery exercises were the backbone of this study and the rationale for their use is discussed in detail in Discussion. They were designed to test two hypotheses: (1) when attention moves in an area of the body, it causes the prana in that area also to move and (2) better movement of prana in an area of the body implies better circulation in that area.  Table 1 outlines the exercises practiced by the yoga group.

Due to the importance of these visualization and guided imagery practices in this study, a brief explanation of them is warranted. In the initial visualization and guided imagery session, the subjects were asked to focus their attention on the place between the nostrils and the upper lip where the air is felt during inhalation and exhalation. In the following visualization and guided imagery sessions, the subjects were asked to visualize the fetus in the uterus and the umbilical cord connecting the fetus to the placenta. Then the participants were guided to visualize healthy blood flow from the mother's heart into the placenta, through the umbilical cord, and bringing nourishment to the fetus.

### 2.7. Data Analysis

For data analysis, PASW Statistics (formerly known as SPSS) version 18.0.3 for Mac was used. Shapiro-Wilk's test was used to test the normality of data. For Doppler and fetal parameters with three measurements in time, repeated measures ANOVA (RM-ANOVA) was performed. However, if the difference between the baseline data of the two groups was statistically significant (fetal heart rate parameter in this study), then ANCOVA test was used, while keeping the baseline data as covariate. When there were only two measurements in time, Independent Samples *t*-test was used for variables that followed a Gaussian distribution at baseline and Mann-Whitney nonparametric test for those that did not. Chi-Square test was used to test significance between groups when frequencies were used.

## 3. Results

### 3.1. Recruitment and Retention

**Figure 1:**
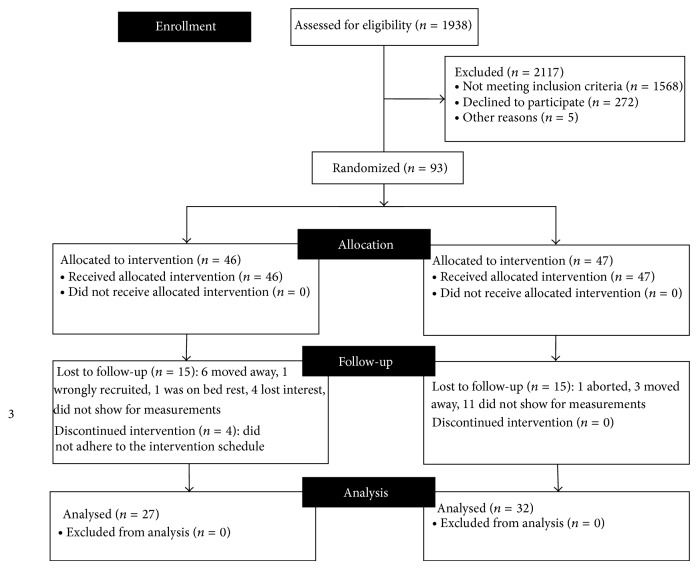
Consort diagram for trial profile.

The consort diagram is presented in Figure 1. There was none with multiple risk factors among the recruited subjects.

### 3.2. Socioeconomic and Demographic Data

A self-reported questionnaire was used to collect demographic data, which included the subjects' age, weight, height, socioeconomics, education, and religion. The financial status of the subjects was measured in two ways: (1) subjectively, by recording the monthly household income (in Indian rupees) reported by the subjects, and (2) objectively, by having the subjects complete a socioeconomic status (SES) form, used by other Indian research groups at SJMCH, which scored the possessions and household features and produced a total score ranging from 0 to 60. These demographic data are listed in Table 2. The majority of the subjects in both groups were between 20 and 35 years of age (only 3 in each group were below 20 years and 1 in the yoga group and 2 in the control group were above 35).

### 3.3. Fetal Measurements

The ultrasound fetal measurements are shown in Table 3. The biparietal diameter, head circumference, femur length, heart rate, and estimated fetal weight showed highly significant improvements in the yoga group (<0.001, 0.002, 0.005, 0.006, and 0.019 *P* values, resp.). As the baseline fetal heart rate (FHR) was significantly different (*P* = 0.036) in the two groups, we used ANCOVA test keeping the baseline values as covariate, which showed significantly lower FHR in the yoga group after 8 weeks (*P* = 0.012) and 16 weeks (*P* = 0.001) of intervention.

### 3.4. Uteroplacental Circulation

Systolic over diastolic ratio (S/D ratio), pulsatility index (PI), resistance index (RI), and diastolic notch were measured in right and left uterine arteries at the 12th, 20th, and 2nd weeks of gestation. These results are listed in Table 4. In the right uterine artery, RI showed significantly less resistance in the yoga group (*P* = 0.01, RM-ANOVA) and near significance results for the PI (*P* = 0.07, RM-ANOVA). In the left uterine artery, near significant result was obtained for RI (*P* = 0.08, RM-ANOVA) and PI (*P* = 0.08, RM-ANOVA). At baseline (12 weeks of gestation), the right uterine artery diastolic notch was detected in 22.6% of the subjects in the yoga group compared to 18.4% in the control group (*P* = 0.67). In the left uterine artery, the percentages were 32.3% and 21.1%, respectively (*P* = 0.29). The number of cases with diastolic notch in the uterine arteries was reduced in both groups as the pregnancy progressed and the interventions were administered. There were much fewer cases in the yoga group compared to the control group, though the differences were not statistically significant.

### 3.5. Fetoplacental Circulation

The S/D ratio, the PI, and the RI parameters of the umbilical and fetal middle cerebral arteries were assessed at the 20th and 28th weeks of gestation through ultrasound Doppler velocimetry. It was not possible to measure these parameters at the 12th week of gestation. All the parameters, except for the RI of the umbilical artery, which showed near significant results, were significantly improved in the yoga group at the 28th week of gestation. All the parameters for the umbilical artery were significantly better in the yoga group even at the 20th week of gestation. The results for the fetoplacental circulation are listed in Table 5. No cases with diastolic notch were detected in either group for umbilical artery or fetal middle cerebral artery.

## 4. Discussion

The arterial resistance index (RI) has been defined to be a measure of pulsatile blood flow that reflects the resistance to blood flow caused by microvascular bed distal to the site of measurement [15]. A resistive index of 0 corresponds to continuous flow; a resistive index of 1 corresponds to systolic but no diastolic flow; and a resistive index greater than 1 corresponds to reversed diastolic flow. Pulsatility index (PI) is a measure of the variability of blood velocity in a vessel, equal to the difference between the peak systolic and minimum diastolic velocities divided by the mean velocity during the cardiac cycle [15]. In contrast, systolic/diastolic (S/D) ratio is a simple ratio of the two. High impedance in the uterine arteries at 20–24 weeks of gestation has been shown to be associated with up to 80% higher risk of developing early onset of preeclampsia [2]. There is also a correlation between RI and development of small-for-gestational-age fetuses [2]. Hence the resistance index (RI) was closely followed up in this study.

This randomized control study on yoga-based visualization and relaxation in high-risk pregnancy has shown significantly better uteroplacental and fetoplacental blood flow velocity in the yoga group compared to the control group. The RI in the right uterine artery was significantly better in the yoga group (*P* = 0.01), while it reached near significance (*P* = 0.08) values for the left uterine artery. Also, the RI in the umbilical artery was significantly better in the study group after 8 weeks of intervention (the 20th week of measurement) and in the fetal MCA after 16 weeks (28th week of measurement) of interventions. Furthermore, significantly fewer occurrences of pregnancy induced hypertension (PIH), preeclampsia, gestational diabetes (GDM), and intrauterine growth restriction (IUGR) cases were observed in the yoga group (*P* = 0.018, 0.042, 0.049, and 0.05, resp.) [12]. Significantly fewer small-for-gestational-age (SGA) babies were born in the study group (*P* = 0.033) [12]. Also, APGAR scores within 1 and 5 minutes of delivery were significantly higher in the yoga group (*P* = 0.006) [12]. As far as the fetal measurements are concerned, there were significant improvements in the biparietal diameter (*P* < 0.001), the head circumference (*P* = 0.002), the femur length (*P* = 0.005), and the estimated fetal weight (*P* = 0.019) in the yoga group.

Interestingly, the umbilical RI was highly significant at the 20th week of measurement (*P* = 0.01) and not significant at the 28th week (*P* = 0.091). The reading may have been influenced by the growing uterus. If so, the increase in MCA flow in the 28th week may indicate that the blood flow to the fetus was still improved in the yoga group although it did not show in the umbilical artery. This hypothesis is further supported by the fact that, in the yoga group, most fetal measurements were significantly improved and significantly fewer complications were observed.

Use of complementary and alternative (CAM) therapies during pregnancy has been on the rise globally [16]. Yoga, due to its ability to lower blood pressure and stress, has been particularly popular [17, 18]. This is important because pharmacological solution for hypertension related complications of pregnancy has shown limited effectiveness in reducing the uterine artery resistance to blood flow [19]. In spite of these findings, clinical research in pregnancy involving CAM therapies are still very few and in between. We were able to find only one Doppler study using yoga interventions, which also reported fewer complications of pregnancy and significantly higher birth weight in the yoga group (*P* < 0.018). However, this study was not randomized and did not report any data on the resistance indices. We could not find any published Doppler study involving tai chi or qi gong in pregnancy. But use of exercise in pregnancy has been widely studied and the overall results support moderate-to-vigorous intensity exercises during pregnancy [20]. Furthermore, it has been shown that exercise in the second half of pregnancy appears to cause a transient increase in the maternal uterine artery pulsatility index without causing any harmful effects on maternal uterine blood flow [21].

Antiplatelet agents, primarily low-dose aspirin [22], and calcium supplementation [23] have been shown to reduce the risk of adverse pregnancy outcomes; however their impact on the uterine artery blood flow is not very clear. Other supplementation, such as the amino acid L-arginine, has been shown to significantly reduce the pulsatility index of the uterine arteries and significantly increase those of the middle cerebral fetal artery and the umbilical artery in women with threatened preterm labor [24].

The sample size for this study is too small to draw any definite conclusion on the mechanism of action of yoga on the reproductive blood flow during pregnancy. Nonetheless, we can examine potential previously argued hypothesis for the results that were observed in this study. Pregnancy itself is a stressful period in a woman's life and it is now believed that it exerts a larger load on the cardiovascular system than previously assumed [25]. In contrast, it is now widely accepted that practices of yoga do reduce stress [26]. Therefore, it is possible that yoga interventions in this study had a positive impact on the maternal stress and have reduced the sympathetic tone, which in turn relaxed the uterine arteries and resulted in a better blood flow. Yoga has been found to decrease blood pressure as well as the levels of oxidative stress in patients with hypertension [27]. This could have led to better trophoblast perfusion and less resistance in the uterine arteries.

Finally, the yoga intervention used in this study was designed with emphasis on the yogic visualization and guided imagery, which, as previously stated, intended to test the hypothesis that when attention is moved to an area of the body, it causes prana to move in that area, which in turn improves circulation in the surrounding tissues. These are not exactly new ideas. Tirumular, an 8th century South Indian saint, once said, “Where the mind goes, the prana follows” [28]. Using ultraviolet photography, it has also been shown that when acupuncture points in a particular meridian are stimulated, the acceleration movement of qi (equivalent to prana in acupuncture [29]) in that meridian results in improved circulation in the tissues surrounding that meridian [29, 30]. But this concept was never investigated scientifically with yoga and certainly not in pregnancy. While the sample size of this study is too small to draw a concrete conclusion, the results point to the important role that yoga can play in high-risk pregnancy.

In our earlier publication, we have shown that the yoga group had lesser number of complications than the control group which could be related to this improved blood flow. Significantly fewer occurrences of pregnancy induced hypertension (*P* = 0.018), preeclampsia (*P* = 0.042), gestational diabetes (*P* = 0.049), and intrauterine growth restriction (*P* = 0.05) were observed in the yoga group. Significantly fewer number had small-for-gestational-age (SGA) babies in the study group (*P* = 0.033) [12]. Also, APGAR scores within 1 and 5 minutes of delivery were significantly higher in the yoga group (*P* = 0.006).

Three participants in the yoga group experienced PIH and none suffered from preeclampsia or eclampsia. In the control group, there were 11 subjects with PIH, 4 with preeclampsia, and 2 with eclampsia [12]. Only one of the four participants with preeclampsia had a uterine artery diastolic notch at the 12th week of Doppler measurement and another at the 20th week of measurement. Therefore, our sample size was not sufficient to detect the predictability of the diastolic notch before 24 weeks of gestation as several other past studies have confirmed.

## 5. Limitations of the Study

The sample size was too small to draw any conclusion on the potential effects of yoga on the diastolic notch of uterine arteries. The high-risk nature of the population for this study contributed to the lower sample size by increase of dropouts due to pregnancy complications. Another reason could have been our strict inclusion criteria that made recruitment more difficult. Furthermore, some of the subjects delivered in their hometowns and we were not able to collect all the necessary data required by the study from the corresponding institutions. This resulted in missing data. In addition, the other hospitals may have used different protocols in delivery, performing C-section or administrating medications during the delivery that could have impacted the outcome data but not the Doppler data that is the focus of this paper. Finally, one of the objectives of this pilot study was to gain knowledge for the design of a larger and more comprehensive follow-up study. We plan to include collection of other parameters, such as gravidity and parity, in the future studies.

## 6. Strengths of the Study

A great deal of efforts was spent in adhering to high standards of randomization and blinding. The data was very carefully entered, double-checked, and analyzed. Also, the sample profile matched closely that of the Bengaluru metropolitan population.

## 7. Future Direction

We recommend a follow-up multicenter RCT with larger sample size powered by the data from this study. We also suggest three groups for such a trial, one control group (walking) and two study groups. One of the study groups will do only the visualizations and guided imagery while the other study group practices the rest of the interventions alone.

## 8. Conclusion

The result of this randomized controlled trial of yoga in high-risk pregnancy has shown that yogic visualization and guided imagery can significantly reduce the impedance in the uteroplacental and fetoplacental circulation. This pilot data can be used to power larger studies to confirm these results and elaborate on the mechanism of action.

## Figures and Tables

**Table 1:** Yoga interventions.

Practices^1^	Duration
Guided relaxation with visualization and imagery	5 min.
Hasta āyama śvasanam (hands in and out breathing)	1 min.
Hastavistāra śvasanam (hands stretch breathing)	2 min.
Gulphavistāra śvasanam (ankles stretch breathing with wall support)	1 min.
Ka*ṭ*iparivartana śvsanam (side twist breathing)	1 min.
Guided relaxation with visualization and imagery	5 min.
Uttānapādāsana śvasanam (leg raise breathing)	1 min.
Setubandhāsana śvasanam (hip raise breathing)	1 min.
Pādasañcālanam (cycling in supine pose)	1 min.
Supta udarākar*ṣ*aṇasana śvasanam (supine abdominal stretch breathing)	1 min.
Vyāghrāsana śvasanam (tiger stretch breathing)	1 min.
Guided relaxation with visualization and imagery	5 min.
Gulphagūraṇam (ankle rotation)	2 min.
Jānuphalakākar*ṣ*aṇam (kneecap contraction)	1 min.
Ardhātitaliāsana (half butterfly exercise)	3 min.
Poornātitaliāsana (full butterfly exercise)	1 min.
Guided relaxation with visualization and imagery	5 min.
Jyotitrā*ṭ*aka (eye exercises)	2 min.
Nā*ḍ*īśuddhi pranayam (alternate nostrils breathing)	2 min.
Deep relaxation in matsyakrī*ḍ*āsana (lateral shavasana)	10 min.

^1^Except for the visualization and guided imagery, all the practices are part of the book [14].

**Table 2:** Demographic data and maternal characteristics at baseline.

	Groups		*P* values
	Yoga (*n* = 24)^c^	Control (*n* = 29)^c^	
Subjects educational profile^1^			
8th grade	1	2	
10th grade	7	5	
12th grade	0	4	*0.19* ^a^
Junior college	0	3	
Bachelor degree	11	11	
Master degree	5	4	
Living arrangement			
Independent^2^	13	13	
With parents	8	13	*0.70* ^a^
With relatives or friends	3	3	
Religion			
Hindu	20	22	
Moslem	0	2	*0.42* ^a^
Christian	4	5	
Age			
Mean (SD)	27.2 (4.8)	27.5 (5.5)	*0.84* ^b^
95% CI	25.1–29.2	25.4–29.5	
Household monthly income^3^			
Mean (SD)	35.4 (28.9)	36.9 (36.4)	*0.87* ^b^
95% CI	22.9–47.8	22.8–51.0	
Socioeconomic^4^			
Mean (SD)	35.4 (7.8)	36.5 (9.4)	*0.67* ^b^
95% CI	32.1–38.7	32.9–40.0	
Maternal weight (kg)			
Mean (SD)	61.8 (13.0)	62.7 (14.6)	*0.82* ^b^
95% CI	56.4–67.3	57.1–68.3	
Maternal height (m)			
Mean (SD)	1.57 (0.05)	1.58 (0.06)	*0.96* ^b^
95% CI	1.55–1.59	1.55–1.59	
Maternal BMI			
Mean (SD)	25.1 (4.8)	25.4 (4.9)	*0.84* ^b^
95% CI	23.1–27.1	23.5–27.2	
Maternal systolic BP			
Mean (SD)	108.3 (12.9)	104.1 (8.3)	*0.18* ^b^
95% CI	102.7–113.9	100.9–107.3	
Maternal diastolic BP			
Mean (SD)	67.5 (9.5)	64.2 (7.6)	*0.18* ^b^
95% CI	63.4–71.6	61.3–67.1	

^1^ *No subject had education below 8th standard*.

^2^Independent: lived with her husband and children, if any.

^3^Family's monthly income in thousands of Indian rupees as reported by the subject.

^4^Socioeconomic status: measured by a standard questionnaire.

^a^ *Calculated using Chi-Square test*.

^b^ *Calculated using Independent Samples t-square test*.

^c^ *There were three subjects in each group that did not complete the demographic questionnaire, which resulted in missing data, hence the lower n values*.

*Remarks: no statistically significant difference was observed between the mean values of socioeconomic parameters of the two groups*.

**Table 3:** Ultrasound fetal measurements between groups.

Parameters	Gestational age	Mean ± SD		*P* values^1^
		Yoga (*n* = 27)	Control (*n* = 32)	
Biparietal diameter (BPD)	12th wk	20.2 ± 4.0	19.5 ± 2.4	***<0.001***
	20th wk	50.6 ± 5.4	46.9 ± 2.4	
	28th wk	72.5 ± 2.9	70.4 ± 2.3	

Head circumference (HD)	12th wk	75.4 ± 9.4	74.3 ± 8.6	***0.002***
	20th wk	181.0 ± 7.9	173.7 ± 7.9	
	28th wk	268.6 ± 8.7	262.4 ± 8.2	

Abdominal circumference (AC)	12th wk	62.3 ± 8.5	60.7 ± 6.2	*0.099*
	20th wk	150.0 ± 10.6	149.1 ± 8.9	
	28th wk	243.7 ± 13.9	236.4 ± 11.1	

Femur length (FL)	12th wk	9.2 ± 1.9	9.3 ± 1.7	***0.005***
	20th wk	33.1 ± 2.1	31.5 ± 1.9	
	28th wk	55.0 ± 2.6	53.0 ± 2.3	

Heart rate (HR)	12th wk	163.5 ± 9.4	157.8 ± 9.4	*0.006* ^a^
	20th wk	149.3 ± 7.6	143.0 ± 9.9	
	28th wk	145.0 ± 11.6	141.3 ± 7.2	

Estimated fetal weight (EFW)	12th wk	0.065 ± 0.02	0.066 ± 0.01	***0.019***
	20th wk	0.362 ± 0.05	0.329 ± 0.04	
	28th wk	1.275 ± 0.15	1.188 ± 0.13	

^1^Calculated using RM-ANOVA.

^a^ANCOVA keeping the baseline data as covariate.

Remarks: significant improvement was observed in all fetal parameters except for AC, which was near significance.

**Table 4:** Measures of uteroplacental circulation between groups.

Arteries		Gestational age	Mean ± SD or count (%)		*P* values
			Yoga (*n* = 27)	Control (*n* = 32)	
Right uterine artery	Systolic/diastolic ratio	12th wk	3.2 ± 1.4	3.1 ± 1.1	*0.17* ^a^
		20th wk	2.3 ± 0.4	2.7 ± 1.1	
		28th wk	2.0 ± 0.3	2.4 ± 0.7	
	Pulsatility index	12th wk	1.4 ± 0.5	1.4 ± 0.5	*0.07* ^a^
		20th wk	0.8 ± 0.2	1.0 ± 0.5	
		28th wk	0.7 ± 0.1	0.9 ± 0.4	
	Resistance index	12th wk	0.65 ± 0.1	0.64 ± 0.1	***0.01*** ^a^
		20th wk	0.52 ± 0.1	0.58 ± 0.1	
		28th wk	0.46 ± 0.1	0.55 ± 0.1	
	Diastolic notch	12th wk	7 (22.6%)	7 (18.4%)	*0.67* ^b^
		20th wk	2 (6.1%)	3 (7.9%)	*0.76* ^b^
		28th wk	1 (3.6%)	1 (2.9%)	*0.87* ^b^

Left uterine artery	Systolic/diastolic ratio	12th wk	3.5 ± 1.5	3.6 ± 1.6	*0.15* ^a^
		20th wk	2.1 ± 0.3	2.6 ± 1.1	
		28th wk	1.9 ± 0.3	2.3 ± 1.2	
	Pulsatility index	12th wk	1.5 ± 0.6	1.5 ± 0.8	*0.08* ^a^
		20th wk	0.8 ± 0.2	1.0 ± 0.5	
		28th wk	0.7 ± 0.1	1.1 ± 1.8	
	Resistance index	12th wk	0.69 ± 0.15	0.66 ± 0.12	*0.08* ^a^
		20th wk	0.52 ± 0.06	0.57 ± 0.11	
		28th wk	0.47 ± 0.07	0.59 ± 0.24	
	Diastolic notch	12th wk	10 (32.3%)	8 (21.1%)	*0.29* ^b^
		20th wk	1 (3.0%)	6 (15.8%)	*0.07* ^b^
		28th wk	1 (3.6%)	3 (8.6%)	*0.42* ^b^

^a^Calculated using RM-ANOVA. ^b^Calculated using Chi-Square test.

Remarks: right uterine artery RI was significantly improved in the yoga group, while the PI was near significance along with the RI and PI of left uterine artery.

**Table 5:** Fetoplacental circulation between groups.

		Gestational age	Mean ± SD		*P* values
			Yoga (*n* = 27)	Control (*n* = 32)	
Umbilical artery	Systolic/diastolic ratio	20th wk	2.7 ± 0.41	3.3 ± 1.1	***0.001*** ^a^
		28th wk	2.6 ± 0.5	2.9 ± 0.6	***0.031*** ^a^
	Pulsatility index	20th wk	1.01 ± 0.18	1.37 ± 0.34	***0.001*** ^b^
		28th wk	0.87 ± 0.18	1.05 ± 0.23	***0.001*** ^b^
	Resistance index	20th wk	0.65 ± 0.05	0.70 ± 0.09	***0.011*** ^b^
		28th wk	0.63 ± 0.08	0.66 ± 0.06	*0.091* ^b^

Fetal middle cerebral artery	Systolic/diastolic ratio	20th wk	5.02 ± 1.47	5.77 ± 2.04	*0.537* ^b^
		28th wk	5.05 ± 1.64	6.62 ± 2.26	***0.01*** ^b^
	Pulsatility index	20th wk	1.86 ± 0.45	2.18 ± 0.67	*0.151* ^b^
		28th wk	1.74 ± 0.53	2.28 ± 1.10	***0.013*** ^b^
	Resistance index	20th wk	0.77 ± 0.07	0.80 ± 0.07	*0.22* ^b^
		28th wk	0.80 ± 0.08	0.85 ± 0.08	***0.048*** ^b^

^a^Calculated using Independent Samples *t*-test.

^b^Calculated using Mann-Whitney test.

Remarks: S/D ratio, PI, and RI parameters of umbilical and fetal middle cerebral arteries were significantly improved in the yoga group after 16 weeks of intervention, except for the RI of umbilical artery, which was near significance.

## References

[1] Khong Y., Brosens I. (2011). Defective deep placentation. *Best Practice and Research: Clinical Obstetrics and Gynaecology*.

[2] Espinoza J., Romero R., Yeon M. K. (2006). Normal and abnormal transformation of the spiral arteries during pregnancy. *Journal of Perinatal Medicine*.

[3] Chaddha V., Viero S., Huppertz B., Kingdom J. (2004). Developmental biology of the placenta and the origins of placental insufficiency. *Seminars in Fetal and Neonatal Medicine*.

[4] Nelissen E. C. M., van Montfoort A. P. A., Dumoulin J. C. M., Evers J. L. H. (2011). Epigenetics and the placenta. *Human Reproduction Update*.

[5] Ross M. G., Beall M. H. (2008). Adult sequelae of intrauterine growth restriction. *Seminars in Perinatology*.

[6] Whitley G. S. J., Dash P. R., Ayling L.-J., Prefumo F., Thilaganathan B., Cartwright J. E. (2007). Increased apoptosis in first trimester extravillous trophoblasts from pregnancies at higher risk of developing preeclampsia. *The American Journal of Pathology*.

[7] Narendran S., Nagarathna R., Nagendra H. R. (2008). *Yoga for Pregnancy*.

[8] Oswal P., Nagarathna R., Ebnezar J., Nagendra H. R. (2011). The effect of add-on yogic prana energization technique (YPET) on healing of fresh fractures: a randomized control study. *Journal of Alternative and Complementary Medicine*.

[9] Sengupta P. (2012). Health impacts of yoga and pranayama: a state-of-the-art review. *International Journal of Preventive Medicine*.

[10] Jayashree R., Malini A., Nagarathna R. (2013). Effect of the integrated approach of yoga therapy on platelet count and uric acid in pregnancy: a multicenter stratified randomized single-blind study. *International Journal of Yoga*.

[11] Babbar S., Parks-Savage A. C., Chauhan S. P. (2012). Yoga during pregnancy: a review. *American Journal of Perinatology*.

[12] Rakhshani A., Nagarathna R., Mhaskar R., Mhaskar A., Thomas A., Gunasheela S. (2012). The effects of yoga in prevention of pregnancy complications in high-risk pregnancies: a randomized controlled trial. *Preventive Medicine*.

[13] Kanako K. (1999). Studies on prophylaxis of preeclampsia by water exercise during pregnancy. *The Journal of the Aichi Medical University Association*.

[14] Narendran S., Nagarathana R., Nagendra H. R. (2010). *Yoga for Pregnancy*.

[15] Deane C., Nikolaides K., Rizzo G., Hecher K., Ximenes R. (2002). Doppler utrasound: principles and practice. *Doppler in Obstetrics*.

[16] Factor-Litvak P., Cushman L. F., Kronenberg F., Wade C., Kalmuss D. (2001). Use of complementary and alternative medicine among women in New York City: a pilot study. *The Journal of Alternative and Complementary Medicine*.

[17] Field T. (2012). Prenatal exercise research. *Infant Behavior and Development*.

[18] Field T. (2011). Yoga clinical research review. *Complementary Therapies in Clinical Practice*.

[19] Khalil A., Harrington K., Muttukrishna S., Jauniaux E. (2010). Effect of antihypertensive therapy with *α*-methyldopa on uterine artery Doppler in pregnancies with hypertensive disorders. *Ultrasound in Obstetrics and Gynecology*.

[20] Szymanski L. M., Satin A. J. (2012). Exercise during pregnancy: fetal responses to current public health guidelines. *Obstetrics and Gynecology*.

[21] Rafla N. M., Etokowo G. A. (1998). The effect of maternal exercise on uterine artery velocimetry waveforms. *Journal of Obstetrics and Gynaecology*.

[22] Roberge S., Nicolaides K. H., Demers S., Villa P., Bujold E. (2013). Prevention of perinatal death and adverse perinatal outcome using low-dose aspirin: a meta-analysis. *Ultrasound in Obstetrics and Gynecology*.

[23] Meads C. A., Cnossen J. S., Meher S. (2008). Methods of prediction and prevention of pre-eclampsia: systematic reviews of accuracy and effectiveness literature with economic modelling. *Health Technology Assessment*.

[24] Rytlewski K., Olszanecki R., Lauterbach R. (2008). Effects of oral L-arginine on the pulsatility indices of umbilical artery and middle cerebral artery in preterm labor. *European Journal of Obstetrics & Gynecology and Reproductive Biology*.

[25] Estensen M. E., Beitnes J. O., Grindheim G. (2013). Altered maternal left ventricular contractility and function during normal pregnancy. *Ultrasound in Obstetrics and Gynecology*.

[26] Curtis K., Weinrib A., Katz J. (2012). Systematic review of yoga for pregnant women: current status and future directions. *Evidence-based Complementary and Alternative Medicine*.

[27] Dhameja K., Singh S., Mustafa M. D. (2013). Therapeutic effect of yoga in patients with hypertension with reference to GST gene polymorphism. *Journal of Alternative and Complementary Medicine*.

[28] Brammarajan A. R. (2000). *Indian Literature: Verses from Patham Thirumurai*.

[29] Nagilla N., Hankey A., Nagendra H. (2013). Effects of yoga practice on acumeridian energies: variance reduction implies benefits for regulation. *International Journal of Yoga*.

[30] Shui Y. L. (2004). *The Biophysics Basis for Acupuncture and Health*.

